# Experimental Study on the Structural Performance of Glass-Fiber-Reinforced Concrete Slabs Reinforced with Glass-Fiber-Reinforced Polymer (GFRP) Bars: A Sustainable Alternative to Steel in Challenging Environments

**DOI:** 10.3390/polym17081068

**Published:** 2025-04-15

**Authors:** Fang Xie, Wanming Tian, Shaofan Li, Pedro Diez, Sergio Zlotnik, Alberto Garcia Gonzalez

**Affiliations:** 1 Department of Civil Engineering, Shaoxing University, Shaoxing 312000, China; tianwanmingsx@163.com; 2 Department of Civil and Environmental Engineering, University of California, Berkeley, CA 94720-1710, USA; shaofan@berkeley.edu; 3 Department of Civil and Environmental Engineering, Universitat Politècnica de Catalunya BarcelonaTech, 08034 Barcelona, Spain; pedro.diez@upc.edu (P.D.);

**Keywords:** glass-fiber-reinforced polymer (GFRP), glass-fiber-reinforced concrete (GFRC) slabs, steel-fiber-reinforced concrete (SFRC), fiber volume fraction (FVF), crack resistance, bearing capacity, ductility, alternative to steel reinforcement, challenging environments

## Abstract

The inherent brittleness of glass-fiber-reinforced polymer (GFRP) bars limits their structural applicability despite their corrosion resistance and lightweight properties. This study addresses the critical challenge of enhancing the ductility and crack resistance of GFRP-reinforced systems while maintaining their environmental resilience. Through experimental evaluation, GFRC slabs reinforced with GFRP bars are systematically compared to steel-reinforced GFRC slabs and non-bar-reinforced SFRC slabs under bending loads. Eight slabs were subjected to four-edge-supported loading following standardized procedures based on prior strength assessments. The results demonstrate that GFRP-reinforced GFRC slabs achieve an ultimate load capacity of 83.7 kN, comparable to their steel-reinforced counterparts (96.3 kN), while exhibiting progressive crack propagation and 17% higher energy absorption than non-fiber-reinforced systems. The load capacity similarities between GFRP-bar-reinforced GFRC slabs and steel-reinforced slabs are 69% for crack loading and 86% for ultimate capacity. Furthermore, this study demonstrates that the reduction factor in flexural strength design of the novel slab should be comprehensively considered, incorporating the recommended value of 0.5. The findings confirm that GFRP-bar-reinforced GFRC slabs meet key structural performance criteria, including enhanced bending capacity, energy absorption, crack resistance, and ductility. This study underscores the potential of GFRP as an effective alternative to steel reinforcement, contributing to the development of resilient and durable concrete structures in demanding environments.

## 1. Introduction

As climate impacts intensify, the demands placed on structural materials by human-engineered environments are becoming increasingly significant [1,2,3]. Extreme weather events, including severe storms, flooding, high humidity, and fluctuating temperatures, are exerting new stresses on buildings, bridges, and infrastructure worldwide. In coastal and humid regions, for instance, materials face accelerated corrosion and degradation, while areas with fluctuating temperatures experience increased risks of cracking due to freeze–thaw cycles.

The early exploration of GFRP in structural applications worldwide began around the 1980s. GFRP, in the 1980s, as a high-performance material, was adopted relatively early in the United States for the reinforcement of highway structures, with research primarily focusing on the application of fiber-reinforced composite bridge decks [4,5,6,7,8]. Around the same period, Miyun, Beijing, China, witnessed the first case of applying fiber-reinforced composites to a highway bridge in 1982 [9]. The bridge had a span of 20.7 m and a width of 9.2 m, constructed with box girders made of honeycomb-structured GFRP. After one year of use, instability in the honeycomb structure and local buckling led to localized depressions in the bridge. In 1987, the GFRP bridge was modified into a GFRP–concrete composite girder, which has since remained in good service [10,11,12,13,14].

While FRP composites were still considered an emerging material in some countries, the British Standard BS 5400-10:1980 [15], published in January 1980, was among the pioneering comprehensive standards. It provided standard load spectra for both highway and railway bridges and recommended methods for assessing the fatigue performance of bridge components subjected to repeated stress fluctuations. In 1992, the Canadian Highway Bridge Design Code (CHBDC, CAN/CSA-S6) incorporated theoretical design methodologies for FRP composites [16]. Subsequently, GFRP underwent rapid development in standardized research, gaining global recognition [17,18,19,20,21]. Researchers worldwide have also made significant contributions to fundamental studies on the use of glass fiber reinforcement to enhance the structural performance of hybrid components in the early stages of development [22,23,24,25,26].

Researchers worldwide are currently focusing on GFRP as a structural material and exploring its various shapes and types [27,28,29,30,31] in engineering composites. Its unique combination of properties makes it ideal for structures subjected to harsh conditions [32,33,34,35,36,37,38,39,40], particularly in challenging environments requiring superior corrosion resistance, insulation, and lightweight materials [41,42,43,44,45,46], supporting the idea that GFRP offers a compelling alternative to traditional steel reinforcement.

**Figure 1:**
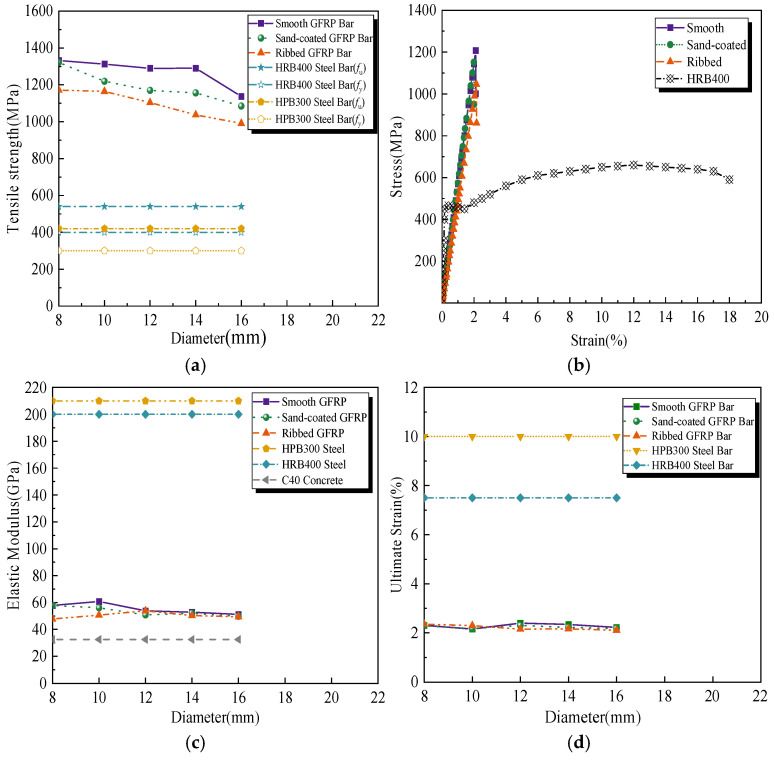
Tensile properties of typical GFRP bars compared with steel bars. Reprinted from Ref. [47]. (**a**) Tensile strength of typical GFRP bars compared with steel bars (**b**) Tensile stress–strain curve of typical GFRP bars compared with steel bars (**c**) Tensile elastic modulus of typical GFRP bars compared with steel bars and (**d**) Tensile ultimate strain of typical GFRP bars compared with steel bars.

As previous studies have shown [47,48], unlike steel reinforcement, which offers a well-defined yield plateau and ductility that enable gradual failure with visible deformation as a warning before collapse, GFRP bars tend to fail abruptly under extreme loads due to their brittle behavior. As [47] shows, as illustrated in Figure 1a,b, while typical GFRP bars have much higher strength than common steel, their tensile failure mode is characterized by explosive brittle fracture. Moreover, unlike steel reinforcement, the tensile stress–strain curve of GFRP bars exhibits a linear distribution without a yield plateau. The lack of ductility of GFRP bars can restrict their use in safety-critical structural applications, particularly in scenarios requiring gradual deformation. Thus, GFRP bars present notable challenges when they are employed without unconfined concrete [48].

Fortunately, when working with GFRP tube-confined concrete, the difference in contributions between GFRP bars and steel bars to the overall hybrid GFRP composite is relatively minor [47,48]. Additionally, a previous study shows that the tensile elastic modulus and ultimate strain of typical GFRP bars are closer to those of conventional concrete compared to steel, as illustrated in Figure 1c,d [47]; this similarity facilitates coordinated deformation between GFRP bars and concrete under loading conditions, forming a robust foundation for the collaborative performance of GFRP-reinforced concrete composite members.

GFRP bars, when integrated with concrete, can exhibit significantly enhanced ductility and crack resistance. As [48] shows, GFRP bars, particularly those with an optimized ribbed surface, an ideal diameter, and paired with a suitable base concrete, can undergo the full bond-slip phase and demonstrate a pronounced yield plateau with visible ductility [48]. This characteristic highlights the potential for exploring GFRP’s structural performance, particularly in hybrid concrete systems under varying conditions, to evaluate its practical applicability in concrete reinforcement.

When combined with appropriate structural configurations, GFRP–concrete beam and column composite elements have gained widespread recognition [49,50,51,52,53,54]. However, research on GFRP integration within composite slab systems remains relatively limited.

This study aims to develop a GFRP-reinforced GFRC slab system, in response to the practical engineering demands for small-sized slabs in civil construction, and assess the compatibility and coordinated performance of all materials within the structural component.

This study aims to evaluate the fundamental structural performance of glass fiber reinforced concrete (GFRC) slabs reinforced with GFRP bars, in comparison to those reinforced with traditional steel bars, which are known for their ductility and crack resistance. Key parameters such as the stress–strain response of the reinforcement bars, load-bearing capacity, crack formation and propagation, and structural ductility are analyzed to assess the feasibility of GFRP bars as a viable alternative to steel in structural applications.

Furthermore, this research provides critical insights into the comparative performance of GFRP and steel reinforcements in GFRC slabs, offering professionals and researchers a clear understanding of their strengths, limitations, and optimal applications. By examining the resilience, brittleness, and crack resistance of GFRC slabs reinforced with GFRP bars, the study contributes to the advancement of hybrid composite materials in civil engineering. Ultimately, it supports evidence-based material selection and structural design strategies for durable, cost-effective, and resilient concrete structures, addressing the growing demand for innovative solutions in challenging environments.

## 2. Materials and Methods

### 2.1. Materials

The materials utilized in this study included GFRP reinforcement bars, sourced from Shandong Safety Industries Co., Ltd., Antai City, China and steel reinforcement bars, supplied by Shanghai Zhongxiang Industrial Co., Ltd., Shanghai, China. The design parameters for both reinforcement bar types, along with their tensile properties measured in accordance with ACI 440.3R-04 [55] and ASTM A370 [56], are summarized in Table 1.

For the comparison of fiber-reinforced concretes, chopped glass fibers and hooked-end steel fibers, commonly employed in engineering applications, were selected. These fibers were provided by Chunfang Mineral Products Co., Ltd., Shijiazhuang City, China and Chang’an Rubber Products Co., Ltd., Hengshui City, China, respectively. Their geometrical shapes and mechanical properties are detailed in Table 2. The fiber volume fractions (FVF) ranged from 1% to 3% for glass fibers and from 0.75% to 3.0% for steel fibers in the GFRC and SFRC strength tests.

The concrete matrix utilized P·O 42.5-grade ordinary Portland cement, produced in accordance with the General Portland Cement standard GB 175 [57]. The fine aggregate consisted of river sand with a fineness modulus of 2.57, while the coarse aggregate comprised crushed stone with a maximum diameter of 12 mm. Table 3 provides the compressive strength and mix proportions for the 36 MPa grade base concrete.

Strength tests for GFRC and SFRC prior to this study were conducted following the Standard for Test Methods of Mechanical Properties on Ordinary Concrete [58]. The fundamental properties of glass-fiber-reinforced (GFRC) and steel-fiber-reinforced concrete (SFRC) are presented in Table 4.

### 2.2. Test Design

The study compares GFRC slabs with a glass fiber volume fraction (FVF) of 3% reinforced with GFRP bars, GFRC slabs with the same FVF reinforced with steel bars, and SFRC slabs with a steel FVF of 3% without reinforcement bars. These slabs, designated as the target slabs shown in columns A to E of Table 5, will be the primary focus of the measurements of the reverent slabs, as shown in columns F to H of Table 5. The FVF parameter selected for the target slabs is based on the mechanical properties of GFRC and SFRC. For GFRC, with FVFs ranging from 1% to 3%, the basic mechanical properties increased consistently with higher FVF. In contrast, for SFRC, with FVFs ranging from 0.75% to 3.0%, the basic mechanical properties showed fluctuations as the FVF increased.

**Figure 2:**
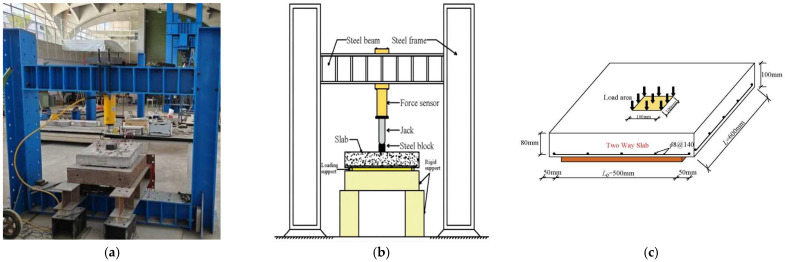

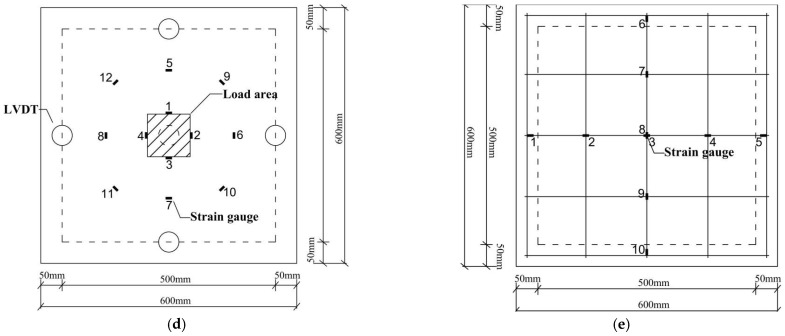
Bending test setup and sample layout. (**a**) Test setup photograph (**b**) Technical diagram of sample arrangement (**c**) Layout of two-way GFRC slab with reinforced bars (**d**) Gauges placed on the slab surfaces to measure concrete strain and (**e**) Gauges located near the bottom of the slabs to measure the reinforced bars strain.

**Figure 3:**
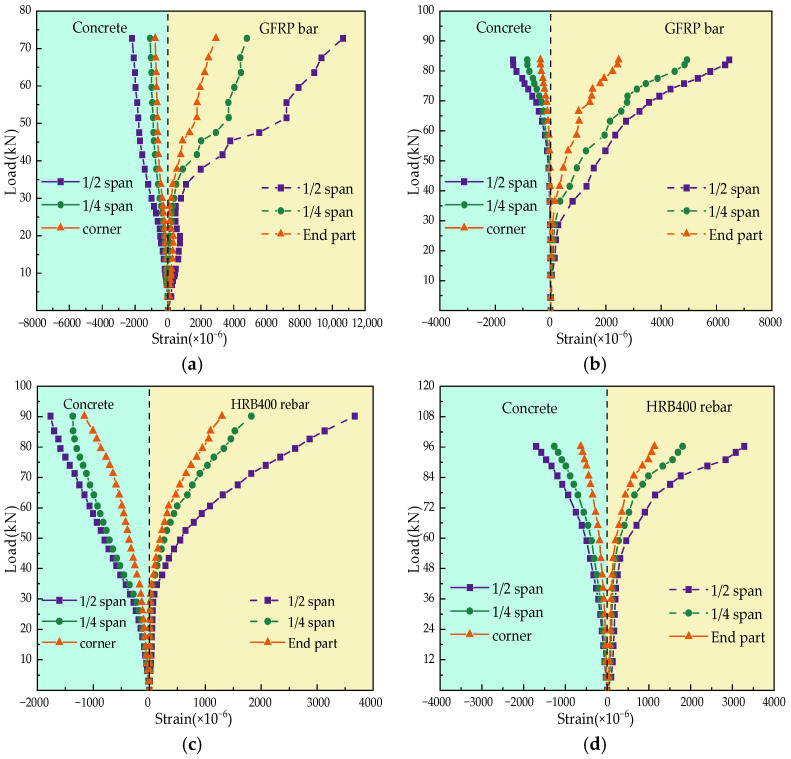

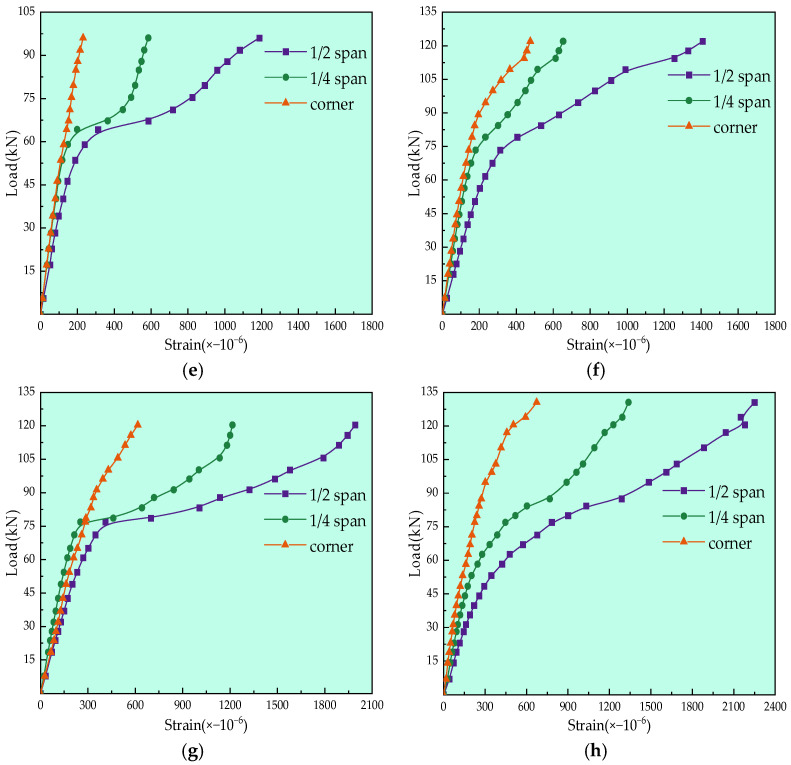
Load vs. strain behavior of concrete and bars in GFRP-bar-reinforced GFRC slabs with 3% FVF. (**a**) GFRC-0-G (**b**) GFRC-3-G (**c**) GFRC-0-S (**d**) GFRC-3-S (**e**) SFRC-0-0 (**f**) SFRC-1-0 (**g**) SFRC-2-0 and (**h**) SFRC-3-0.

All slabs were tested using a 50-ton reaction frame system. Figure 2a illustrates the bending test setup, while Figure 2b shows the sample arrangement. Each slab is square, with a side length of 600 mm, a thickness of 100 mm, and an effective span of 500 mm. The reinforcement bars in the GFRC slabs, as shown in Figure 2c, are spaced 140 mm apart. Strain gauges, each 50 mm in length, were placed on the slab surfaces to measure concrete strain, while 3 mm gauges measured strain in the reinforcements (either GFRP or steel bars) located near the bottom of the slabs, as shown in Figure 2d and Figure 3e. A dial gauge with a 50 mm range was positioned at the center of the bottom of each slab. The loading procedure followed the guidelines in the Standard for Testing Methods of Concrete Structures [59].

## 3. Results

### 3.1. Load-Strain Behavior

Strain measurements were conducted on the concrete and reinforcement bars of the fiber-reinforced slabs under bending load, as shown in Figure 3. Figure 3a–d illustrate the strain developments in GFRP-bar-reinforced GFRC slabs compared to steel-bar-reinforced GFRC slabs, where the development of concrete strains in GFRC slabs is relatively similar, particularly in terms of their ultimate values. It can be observed that the strains in the top concrete of SFRC slabs are significantly smaller than those in GFRC slabs, as shown in Figure 3e–h. This is mainly because glass-fiber-reinforced employs a lower elastic modulus than steel-fiber-reinforced concrete.

Meanwhile, the strain in GFRP reinforcement is significantly greater than that in steel reinforcement, as illustrated in Figure 3. This is mostly attributed to the mechanical properties of GFRP bars, which are characterized by high strength and low elastic modulus. However, GFRP bars typically do not reach their ultimate tensile strength during flexural loading, and it is difficult to achieve their limited strains because the deformation of the hybrid system in cooperation with the concrete is restricted. In contrast, steel reinforcements generally reach their yield strains (approximately 0.002 at the mid-span for steel bar HRB 400), where they sufficiently contribute their flexural load capacity. Therefore, the design of GFRP reinforcements in glass-fiber-reinforced slabs typically differs from that of steel-reinforced slabs. The structural performance of flexural components should be comprehensively considered to prevent potential excessive strain and subsequent safety risks.

### 3.2. Load-Deflection Behavior

While defining the crack load and ultimate load of SFRC-3-N slab as F^*^_cr_ and F^*^_u_, respectively, the values of *F*_cr_/F^*^_cr_ and *F*_u_/F^*^_u_ for each slab type were obtained, as shown in Table 6. The values of *F*_cr_/F^*^_cr_ and *F*_u_/F^*^_u_ in GFRP-bar-reinforced GFRC slabs were found to be significantly lower than those in steel-bar-reinforced GFRC slabs. The differences in load capacity under bending conditions among different types, GFRP-bar-reinforced GFRC slabs, steel-bar-reinforced GFRC slabs, and non-bar-reinforced SFRC slabs are limited to a certain range, as demonstrated by the ratio ranges of 0.5–1 for *F*_cr_/F^*^_cr_ and 0.6–1 for *F*_u_/F^*^_u_.

Notably, when comparing the ratio of crack load to ultimate load capacity (*F*_cr_/*F*_u_) among these three types of slabs, as shown in Table 6, the ratio values for each slab type are relatively close. The *F*_cr_/*F*_u_ value of GFRC-3-G is 0.44, which is closer to that of GFRC-0-G (0.42) than to SFRC-3-N (0.51). Similarly, the *F*_cr_/*F*_u_ value of GFRC-3-S (0.54) is slightly closer to that of GFRC-0-S (0.52) than to SFRC-3-N (0.51). This observation highlights the slight differences when comparing the ratio of the crack load to the ultimate load capacity (*F*_cr_/*F*_u_) under bending among GFRP-bar-reinforced GFRC slabs (0.44), steel-bar-reinforced GFRC slabs (0.54), and non-bar-reinforced SFRC (0.51) with the same fiber volume fraction (FVF).

Interestingly, the ratio of mid-span deflection at the crack load to that at the ultimate load (*δ*_cr_/*δ*_u_) is also quite similar across the same slab type. The *δ*_cr_/*δ*_u_ value of GFRC-3-G is 0.14, which is much closer to that of GFRC-0-G (0.11) than to SFRC-3-N (0.12). Similarly, the *δ*_cr_/*δ*_u_ value of GFRC-3-S (0.32) is much closer to that of GFRC-0-S (0.31) than to SFRC-3-N (0.12). Notably, the ratios of mid-span deflections at crack load and ultimate load, the value *δ*_cr_/*δ*_u_, in GFRP-reinforced GFRC slabs are much closer to those in SFRC slabs than to steel-reinforced GFRC slabs. It shows relative differences in deflection behavior between GFRP-bar-reinforced and steel-bar-reinforced GFRC slabs compared to SFRC with a 3% FVF, as shown in Table 6.

## 4. Discussion

### 4.1. Crack Propagation and Fiber Contribution

The bending test results of fiber-reinforced slabs highlight the contributions of the fibers. Both GFRC and SFRC slabs demonstrate significant enhancements in crack load capacity and ultimate load capacity when compared to non-fiber-reinforced concrete. These improvements are evident from the data presented in Table 6. Specifically, in GFRC slabs reinforced with glass-fiber-reinforced polymer (GFRP) bars and incorporating a 3% fiber volume fraction (FVF), the crack load and ultimate load capacity increased by 21% and 17%, respectively, relative to GFRP-reinforced slabs without fiber reinforcement. Similarly, in GFRC slabs reinforced with steel reinforcement and a 3% FVF, the crack load and ultimate load capacity exhibited increases of 15% and 10%, respectively, compared to steel-reinforced slabs without fiber reinforcement.

**Figure 4:**
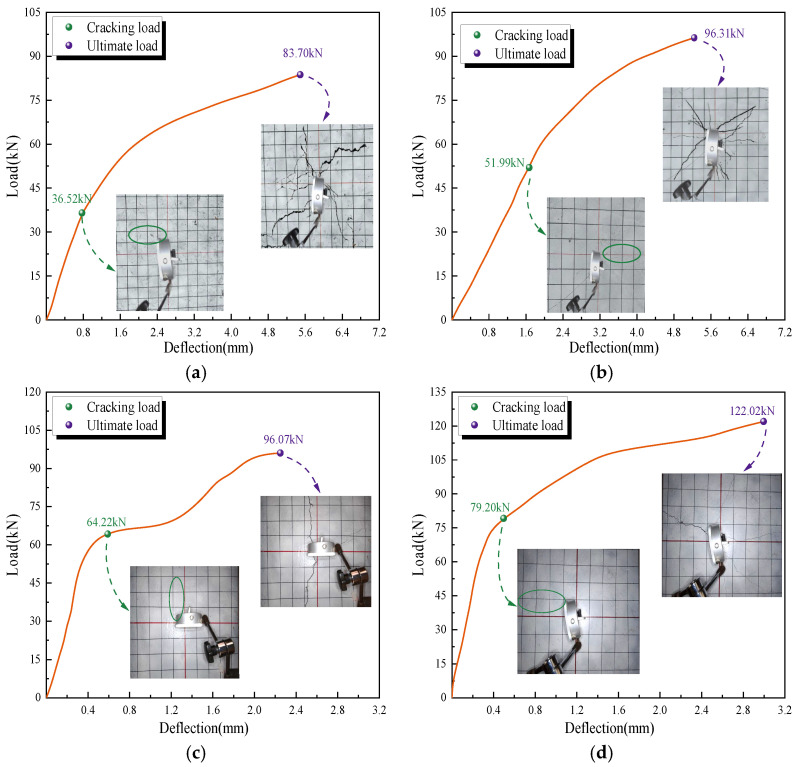

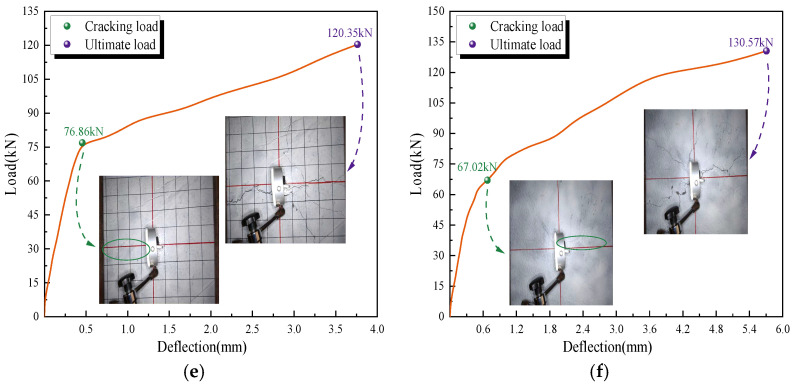
Crack propagation of GFRP-bar-reinforced GFRC slabs compared to steel-bar-reinforced GFRC slabs and non-bar-reinforced SFRC slabs. (**a**) GFRC with FVF 3% and GFRP bar reinforcement (GFRC-3%-G) (**b**) GFRC with FVF 3% and steel bar reinforcement (GFRC-3%-S) (**c**) SFRC with FVF 0.75% and no bar reinforcement (SFRC-0-N) (**d**) SFRC with FVF 1.5% and no bar reinforcement (SFRC-1-N) (**e**) SFRC with FVF 2% and no bar reinforcement (SFRC-2-N) and (**f**) SFRC with FVF 3% and no bar reinforcement (SFRC-3-N).

Figure 4 illustrates the crack propagation in reverent slabs of GFRP-bar-reinforced GFRC slabs, steel-bar-reinforced GFRC slabs, and non-bar-reinforced SFRC slabs. It demonstrates that glass fibers help distribute stress by delaying and preventing the formation of larger cracks. Both GFRP-bar-reinforced GFRC slabs and steel-bar-reinforced GFRC slabs, compared to SFRC slabs with a 3% FVF, exhibit relatively gradual load-deflection response and predictable crack propagation, with a certain difference in load capacity, as demonstrated in Table 6 and illustrated in Figure 4a,b,f. Notably, SFRC slabs exhibit fluctuations in bending capacity as the FVF increases, aligning with the typical performance of SFRCs, whereas GFRCs demonstrate consistent and monotonic improvements with increasing FVF.

### 4.2. Design of Flexural Strength

The design flexural strength at a section of a GFRP-bar-reinforced slab must exceed the factored moment to comply with ACI provisions [20]. The design flexural strength is defined as the nominal flexural strength of the member multiplied by the strength reduction factor, as shown in Equation (1).

(1) $$M_{u}\leq \Phi M_{n}$$

Further calculations can be performed using Equations (2) and (3), as shown below, to determine the nominal flexural strength.

(2) $$M_{n}=\rho_{f}\cdot f_{f}(1-0.59\frac{\rho_{f}\cdot f_{f}}{f_{c}^{\prime }})bd^{2}$$

(3) $$f_{f}=\sqrt{\frac{{\left(E_{f}\varepsilon_{cu}\right)}^{2}}{4}+\frac{0.85\beta_{1}f_{c}^{\prime }}{\rho_{f}}E_{f}\varepsilon_{cu}}-0.5E_{f}\varepsilon_{cu}$$

Meanwhile, the strength reduction factor can be determined [20] using Equation (4) as a function of the reinforcement ratio, which can be obtained using Equations (5) and (6).

(4) $$\Phi =\left\{\begin{array}{c} 0.55,\rho_{f}\leq \rho_{fb}\\ 0.3+\frac{\rho_{f}}{4\rho_{fb}},\rho_{fb}<\rho_{f}<1.4\rho_{fb}\\ 0.65,\rho_{f}\geq 1.4\rho_{fb} \end{array}\right.$$

(5) $$\rho_{f}=A_{f}/bd$$

(6) $$\rho_{fb}=0.85\beta_{1}\frac{f_{c}^{\prime }}{f_{fu}}\cdot \frac{E_{f}\varepsilon_{cu}}{E_{f}\varepsilon_{cu}+f_{fu}}$$

By applying the aforementioned equations and conducting a comparative analysis with the JSCE 1997 [21] recommendation, the design flexural strength and strength reduction factor for GFRP-bar-reinforced slabs, steel-bar-reinforced slabs, and SFRC slabs are determined, as summarized in Table 7.

It can been observed in Table 7, the nominal flexural strength of GFRP-bar-reinforced slabs (0.59 for 3% FVF) is observed to be slightly lower than that predicted by the ACI provision (0.65). Meanwhile, the nominal flexural strength of steel-bar-reinforced slabs (1.04 for 3% FVF) is slightly higher than the corresponding value (0.9) specified by the ACI provision.

Therefore, the flexural strength of our novel system can be analyzed in accordance with the provisions of the ACI standard. However, the design flexural strength must be comprehensively evaluated by considering the critical strength reduction factor, which is hereby recommended to be 0.50 for GFRP-bar-reinforced GFRC slab system.

Notably, it can be observed that the fiber-reinforced contribution in both GFRP-bar-reinforced GFRC slabs and steel-bar-reinforced GFRC slabs can be quantified by calculating the design flexural strength at 3% FVF, which results in increases of 16.96% and 6.47%, respectively, compared to non-fiber-reinforced slabs. These improvements suggest that the hybrid system may provide additional benefits due to the synergistic effect of multiple reinforcement materials, thereby enhancing the overall flexural capacity and ductility of the component, which in turn contributes to improved structural performance in practical applications.

### 4.3. Hybrid Engineering Performance

The engineering performance of hybrid components is influenced by multiple parameters, leading to complex interactions that directly affect key structural properties such as bearing capacity, ductility, and energy absorption. These interactions ultimately contribute to a more ductile, resilient, and predictable structural failure behavior.

The structural performance of hybrid slabs, including bearing capacity, ductility, and energy absorption, can be evaluated through analytical formulations. Specifically, the bearing capacity can be determined based on the transfer of flexural strength, as expressed in Equations (1)–(6) in Section 4.2.

Ductility for flexural members is quantified as the ratio of deflections at the ultimate load to those at the crack load, as shown in Equation (7). Meanwhile, toughness, which represents a slab’s ability to absorb energy and resist fracture during crack propagation before bending failure, is typically assessed by integrating the critical load-deflection curve, as experimentally derived, shown in (8).

(7) $$\mu =\delta_{u}/\delta_{y}$$

(8) $$T=\int_{0}^{\delta_{u}}P(\delta )d\delta$$

A comprehensive performance in typical hybrid GFRP-bar-reinforced GFRC slabs compared to steel-bar-reinforced GFRC slabs and non-bar-reinforced SFRC slabs is obtained, comprising predictive indicators for engineering, as shown in Table 8. The results demonstrate that GFRP-bar-reinforced GFRC slabs meet several critical structural performance criteria, including bending capacity, energy absorption, crack resistance, structural ductility, and safety warning, in comparison to steel-reinforced slabs. Moreover, these slabs present significant advantages such as corrosion resistance, cost-effectiveness, ease of processing, insulation properties, and water resistance—factors that are crucial for enhancing the engineering resilience of structures exposed to diverse climatic conditions.

## 5. Conclusions

This study systematically evaluates the structural performance of glass-fiber-reinforced (GFRC) slabs reinforced with GFRP bars, comparing them to steel-reinforced GFRC slabs and non-bar-reinforced steel-fiber-reinforced concrete (SFRC) slabs under bending loads. The results demonstrate that the GFRP-reinforced GFRC slab system maintains the compatibility and coordinated performance of all materials within the structural component. The findings confirm that GFRP-bar-reinforced GFRC slabs satisfy key structural performance criteria, including improved bending capacity, energy absorption, crack resistance, and ductility. The main conclusions are as follows:

(1) The enhancement of concrete performance through the incorporation of fibers is particularly evident. GFRP-bar-reinforced GFRC slabs with 3% FVF, similar to steel-bar-reinforced GFRC slabs and SFRC slabs, exhibit a gradual load-deflection response and predictable crack propagation.
(2) The differences in load capacity under bending conditions between GFRP-bar-reinforced GFRC slabs and steel-reinforced slabs are limited to a specific range. The similarities in load capacities are indicated by ratio values of 70% for crack load capacity and 86% for ultimate load capacity, respectively.
(3) GFRCs exhibit consistent and monotonic improvements in flexural strength with increasing FVF. The flexural strength can be analyzed according to the provisions of the ACI standard, taking into account the strength-reduction factor, which is recommended to be 0.5.
(4) When analyzed using current provisions, the hybrid system may offer additional benefits due to the synergistic effect of multiple reinforcement materials, enhancing the overall flexural capacity and ductility of the component, thereby improving structural performance in practical applications.
(5) As demonstrated, the novel system significantly enhances bending capacity, crack resistance, energy absorption, structural ductility, and safety indicators compared to traditional steel-reinforced slabs. These enhancements are crucial for increasing the structural resilience of buildings exposed to diverse climatic conditions and will support the follow-up study.

## Figures and Tables

**Table 1:** Geometrical shapes and tensile properties of GFRP and steel reinforcement bars.

Bar Type	Photograph	Geometry	Length (mm)	Diameter (mm)	Tensile/Yield Strength (MPa)	Elastic Modulus (GPa)	Density (10^3^ kg/m^3^)
GFRP	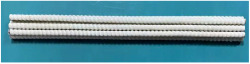	Threaded	560	8	1172 ± 39	48 ± 0.95	2.85
Steel (HRB400)	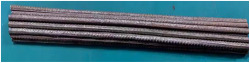	Threaded	560	8	400 ± 13	210 ± 5.46	7.85

**Table 2:** Geometrical shapes and mechanical properties of glass and steel fibers.

Fiber Type	Photograph	Length (mm)	Diameter (mm)	Aspect Ratio	Tensile Strength (MPa)	Elastic Modulus (GPa)	Density (10^3^ kg/m^3^)
Glass Fiber	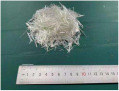	18	16	1.125	600	45	2.70
Steel Fiber	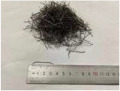	35	0.5	70.00	800	200	7.85

**Table 3:** Compressive strength and mix proportions of base concrete.

Base Concrete	Compressive Strength (MPa)	Fine Aggregate (kg/m^3^)	Coarse Aggregate (kg/m^3^)	Cement (kg/m^3^)	Water (kg/m^3^)
C36	35.51	580.60	1281.40	434.20	128.80

**Table 4:** Fundamental properties of glass-fiber-reinforced (GFRC) and steel-fiber-reinforced concrete (SFRC).

ID	Fiber	FVF (%)	Compressive Strength (MPa)	Compressive Failure and Crack Propagation	Tensile Strength (MPa)	Tensile Failure and Crack Propagation
GFRC-0	None	0	35.51 ± 0.78	Conventional concrete exhibits fully and abruptly split along the crack without prior warning or visible indications.	3.24 ± 0.10	Conventional concrete forms sudden and complete splitting along the crack without visible indication.
GFRC-1	Glass	1.0	35.84 ± 1.65	Glass fiber reinforced with insufficient fiber volume performs, to a large extent, similarly to conventional concrete. Glass fiber reinforced with sufficient fiber volume develops cracks progressively with gradual crushing, accompanied by reduced spalling.	3.39 ± 0.14	Glass fiber reinforced with insufficient fiber volume performs close to conventional concrete. Glass fiber reinforced with sufficient fiber volume eventually splits through cracks that develop progressively.
GFRC-2		2.0	36.65 ± 0.88		3.44 ± 0.14	
GFRC-3		3.0	38.76 ± 1.28		3.67 ± 0.13	
SFRC-0	Steel	0.75	40.03 ± 1.08	Steel-fiber-reinforced concrete forms cracks more gradually, maintaining structural integrity without shattering.	5.16 ± 0.26	Steel-fiber-reinforced concrete develops cracks slowly and subtly, ultimately preventing splitting and preserving structural integrity.
SFRC-1		1.5	40.60 ± 0.93		5.82 ± 0.23	
SFRC-2		2.0	48.60 ± 2.04		4.12 ± 0.08	
SFRC-3		3.0	37.98 ± 1.41		4.16 ± 0.11	

**Table 5:** Design parameters of GFRP-bar-reinforced GFRC slabs compared to steel-bar-reinforced GFRC slabs and non-bar-reinforced SFRC slabs.

Target Slab ID	Fiber	FVF (%)	Reinforced Bars	Relevant Slab	Relevant FRC	Fiber	FVF (%)
GFRC-3-G	Glass	3.0	GFRP	GFRC-0-G	GFRC-0	Glass	0
				GFRC-3-G	GFRC-1		1
GFRC-3-S		3.0	Steel	GFRC-0-S	GFRC-2		2
				GFRC-3-S	GFRC-3		3
SFRC-3-N	Steel	3.0	None	SFRC-0-N	SFRC-0	Steel	0.75
				SFRC-1-N	SFRC-1		1.5
				SFRC-2-N	SFRC-2		2.0
				SFRC-3-N	SFRC-3		3.0

**Table 6:** Load-deflection behavior of GFRP-bar-reinforced GFRC slabs compared to steel-bar-reinforced GFRC slabs and non-bar-reinforced SFRC slabs.

Slab ID	Fiber	FVF/%	Bar	*F*_cr_/kN	*F*_cr_/F^*^_cr_	*F*_u_/kN	*F*_u_/F^*^_u_	*F*_cr_/*F*_u_	*δ*_cr_/mm	*δ*_cr_/δ^*^_cr_	*δ*_u_/mm	*δ*_u_/δ^*^_u_	*δ*_cr_/*δ*_u_
GFRC-0-G	Glass	0	GFRP	30.07	0.45	71.37	0.55	0.42	0.66	0.99	5.85	1.02	0.11
GFRC-3-G		3		36.52	0.54	83.70	0.64	0.44	0.77	1.15	5.49	0.96	0.14
GFRC-0-S		0	Steel	45.37	0.68	87.88	0.67	0.52	1.62	2.42	5.23	0.92	0.31
GFRC-3-S		3		51.99	0.78	96.31	0.74	0.54	1.67	2.50	5.24	0.92	0.32
SFRC-0-N	Steel	0.75	None	64.22	0.96	96.07	0.74	0.67	0.59	0.88	2.25	0.39	0.26
SFRC-1-N		1.5		79.20	1.18	122.02	0.93	0.65	0.50	0.75	3.00	0.53	0.17
SFRC-2-N		2		76.86	1.15	120.35	0.92	0.64	0.45	0.67	3.76	0.66	0.12
SFRC-3-N		3		67.02	1	130.57	1	0.51	0.67	1	5.71	1	0.12

**Table 7:** Design flexural strength and the reduction factor of GFRP-bar-reinforced GFRC slabs.

**Slab ID**	*Mn* (kN · m)	*M*_u_ (kN · m)	ACI 440.1R [20]		JSCE 1997 [21]		*Φ*	*c_f_* (%)
			*Φ*_theo1_	*M*_utheo1_ (kN · m)	*Φ*_theo*2*_	*M*_utheo2_ (kN · m)		
GFRC-0-G	8.606	4.475	0.65	5.594	0.77	6.627	0.52	-
GFRC-3-G	8.871	5.234	0.65	5.766	0.77	6.861	0.59	16.96
GFRC-0-S	5.756	5.641	0.9	5.180	0.9	5.180	0.98	-
GFRC-3-S	5.775	6.006	0.9	5.198	0.9	5.198	1.04	6.47

**Table 8:** Structural performance of GFRP-bar-reinforced GFRC slabs compared to steel-bar-reinforced GFRC slabs and non-bar-reinforced SFRC slabs.

ID	Bearing Capacity/kN		Ductility /*μ*	Toughness/kN · mm	Bending Capacity	Energy Absorption	Crack Resistance	Corrosion Resistance	Structural Ductility	Safety Warning
	Crack	Ultimate								
GFRC-0-G	30.07	71.37	2.57	310.40						
GFRC-3-G	36.52	83.70	4.80	332.75	√	√	√	√	√	√
GFRC-0-S	45.37	87.88	1.44	292.80						
GFRC-3-S	51.99	96.31	3.55	330.69	√	√	√		√	√
SFRC-0-N	64.22	96.07	1.65	153.05						
SFRC-1-N	79.20	122.02	2.50	291.56						
SFRC-2-N	76.86	120.35	3.25	344.99						
SFRC-3-N	67.02	130.57	5.05	565.57	√	√	√		√	√

Note: Select ‘√’ as best in the same type slab team.

## Data Availability

The authors confirm that the data supporting the findings of this study are available within the article.
